# Prevalence of and factors associated with multimorbidity among adults in Kuwait

**DOI:** 10.1186/s12889-024-18298-z

**Published:** 2024-03-12

**Authors:** Fajer Saoud, Maryam AlHenaidi, Hajer AlOtaibi, Asayel AlEnezi, Mariam Mohammed, Fatemah AlOtaibi, Danah AlShammari, Sebakah AlKharqawi, Hadil AlMayas, Hatoun AlMathkour, Saeed Akhtar

**Affiliations:** Department of Community Medicine and Behavioural Sciences, College of Medicine, Kuwait University, PO Box 24923, 13110 Safat, Kuwait

**Keywords:** Multimorbidity, Prevalence, Risk factors, Polychotomous logistic regression, Kuwait

## Abstract

**Background:**

This cross-sectional study aimed to assess the prevalence of morbidity *i.e.,* one morbidity and multimorbidity (≥ 2 morbid conditions) among adults in Kuwait and to examine the sociodemographic and lifestyle factors associated with morbidity as a multinomial outcome in the study population.

**Methods:**

The data were collected from January 26, 2021, to February 3, 2021, using an electronic questionnaire, which was distributed on social media platforms. The consent form was attached with the questionnaire and the participants were requested to sign the consent form before completing the questionnaire. The prevalences (%) of each morbidity and multimorbidity were computed. Multivariable polychotomous logistic regression analysis was used to evaluate the association between the demographic and lifestyle factors with morbidity as a multinomial outcome.

**Results:**

Of 3572 respondents included, 89% were Kuwaiti, 78.3% females and 66% were 21- 40 years old. The prevalence of multimorbidity and one morbidity respectively was 27.4% and 29.7%. The prevalence of multimorbidity with two, three, four or five ill-health conditions were 14.3%, 7.4%, 3.5%, and 1.2%, respectively. A higher prevalence of multimorbidity was among respondents over 60 years of age (71%) and Kuwaiti nationals (28.9%). The final multivariable polychotomous logistic regression model revealed that age, sex, nativity, sedentary lifestyle, smoking, and alcohol drinking were significantly (*p* < 0.05) associated with multimorbidity. However, age and alcohol drinking were significant (*p* < 0.05) predictors of one morbidity.

**Conclusion:**

This study provides evidence that multimorbidity is more prevalent among the elderly, females, and Kuwaiti nationals. Sedentary behaviour, smoking and alcohol consumption were significantly and independently associated with multimorbidity. These findings highlight the burden of multimorbidity and should be considered in the development of future prevention programs.

**Supplementary Information:**

The online version contains supplementary material available at 10.1186/s12889-024-18298-z.

## Introduction

As worldwide life expectancy increases from the epidemiologic transition, the incidence of non-communicable diseases and multimorbidity tends to increase as well. The World Health Organization (WHO) has defined multimorbidity, or multiple chronic conditions (MCC), as the co-occurrence of two or more long-term medical conditions in an individual, where one is not necessarily more central than the other [1, 2]. This contrasts with comorbidity, where one or more additional conditions co-occur with the presence of a primary condition as an index disease [3]. A systematic review has highlighted three definitions of multimorbidity from three different perspectives: epidemiology and public health, clinicians and patients in daily clinical practice, and long-term care and family medicine in primary care denoting its complexity [1]. However, the most adopted definition of multimorbidity is that of the WHO [1, 4, 5]. The umbrella of multimorbidity includes physical and long-term mental conditions of both low and high prevalence. Published data demonstrated three distinct multimorbidity patterns: cardiometabolic, mental health, and musculoskeletal problems [1].

Challenges of multimorbidity not only stress the health-care system but also largely impact the patient as an individual. One of the most significant challenges is polypharmacy, which results in several complications including adverse drug interactions and poor compliance with the extensive list of prescribed medications [2, 6]. It is also associated with an increased burden on health-care utilization and cost, where 66% of total health-care spending is directed toward care for approximately 27% of patients with MCC [7]. Higher risk of mortality, poor functional status, increased hospitalization, and conflicting medical advice are additional challenges associated with multimorbidity [7].

In the United States, one in four adults has two or more concurrent chronic conditions [8]. In Scotland, more than 23% of the population had two or more long-term conditions [9]. A cross-sectional study showed an increasing level of multiple chronic conditions in low- and middle-income countries [10]. In the Middle East, noncommunicable diseases account for 53% of annual mortality with 2.3 million deaths per year; this increasing trend in the prevalence of chronic diseases has led to a more serious issue of multimorbidity in the region [11]. A recent retrospective cohort study showed a 21.1% prevalence of multimorbidity in the Iranian population [12]. In Saudi Arabia, one in five diabetics reported having two or more comorbidities [13]. In Kuwait, a study conducted a decade ago, reported a high prevalence of comorbidities of hypertension, diabetes, and heart disease among Kuwaitis aged 50–59 years (9.4%) and 60–69 years (20.9%) [14].

Published literature has shown a significant association between multimorbidity and multiple sociodemographic factors. For instance, the prevalence of multimorbidity increases substantially with age; the most common type of multimorbidity in those aged 55 and above is purely physical, whereas the younger age group is more likely to experience a mixed physical and mental health related morbidities [15]. Moreover, within an individual disease clusters differ by sex; cardiometabolic disorders are more prevalent in men, while psychogeriatric and mental health patterns are more prevalent in women [16]. In addition, the burden of MCC is more pronounced in poorer communities [17]. Multimorbidity is also associated with multiple lifestyle behaviors such as smoking, low physical activities, dietary pattens, and others [4].

Research regarding multimorbidity is still at its earliest stages, specifically in Kuwait; a country with a population of 4.7 million with 70% being expatriates and an estimated life expectancy of 75.4 years. As opposed to available data regarding the prevalence of individual chronic diseases in Kuwait, few published data have focused on the co-occurrence of these long-term illnesses. A study that includes a wider range of morbidities that are highly prevalent in Kuwait and a wider range of age groups is a crucial first step to fully comprehend the multimorbidity phenomena in the target population. Therefore, this cross-sectional study aimed to estimate the prevalence of multimorbidity (2 or more ill-health conditions) among adults and examine the sociodemographic and lifestyle factors associated with multimorbidity in the study population.

## Methods

### Study design, setting, and participants

This cross-sectional study was undertaken during January 2021 in the adult population of Kuwait. Individuals 21 years old or older, of either sex (male or female), any nationality (Kuwaiti nationals, non-Kuwaiti Arabs, or non-Kuwaiti non-Arabs), residing in any of the six governorates of Kuwait, who can read and write English and/or Arabic languages were eligible to participate in the study. However, short-term visitors of Kuwait were excluded from participation.

### Questionnaire

For data collection, a self-administered and structured electronic questionnaire was developed in both English and Arabic. Based on the relevant literature review, our questionnaire included 36 questions composed of dichotomous (yes/no) as well as multiple-choice questions [15, 16]. The questionnaire comprised three sections, which are briefly summarized below.

### Sociodemographic factors

This section comprised questions on age (completed years), sex, and nationality (Kuwaiti, non-Kuwaiti Arab, and non-Kuwaiti non-Arab). In addition, the participants were asked about their educational level (less than high school, high school, college, and postgraduate education), total family income in Kuwaiti Dinars (KD) per month (less than 500, 500–900, 1000–1499, 1500–2000, and more than 2000) and marital status (single, married, divorced, and widowed).

### Multimorbidity assessment

Multimorbidity was defined according to the WHO as the coexistence of two or more chronic conditions in an individual [2]. Twelve distinct morbidities were assessed by asking questions to confirm whether the participant had ever been told by a physician that he/she had any of the 12 health problems [18]. Two online databases were reviewed (PubMed and Scopus) for published evidence on chronic multimorbidity worldwide, in the Middle East, and Kuwait. To the best of our knowledge, published literature on the ranking of incident or prevalent comorbidities in Kuwait was lacking. Thus, an overview of 53 systematic reviews was used to compile a list of 12 common morbidities including hypertension, cardiovascular diseases, diabetes, chronic obstructive pulmonary disease, asthma, osteoarthritis, rheumatism, osteoporosis, depression, stroke, and cancer [1].

### Lifestyle factors

Questions on the following lifestyle factors were included: smoking (dose, duration and methods of smoking), physical activity and sedentary behaviour. There were also questions on dietary patterns (fruit and vegetable intake, fast food, seafood, and red meat) and alcohol consumption. Smokers were categorized into three categories: light, moderate or heavy smokers [19]. WHO guidelines were used to assess physical activity and sedentary behaviour [20]. A short dietary assessment method, also known as dietary screener, was used to assess the dietary habits of the participants [21]. The participants were categorized based on alcohol consumption status as: lifetime abstainers, past 12 months abstainers or current drinkers [22].

### Data collection and sample size

Due to the Kuwait Ministry of Health (MOH) regulations to control the SARS-CoV-2 pandemic, the data were collected via an online questionnaire using Google forms program. The questionnaire was distributed by the members of the research team via social media platforms (Twitter, Instagram, WhatsApp, Snapchat, and Facebook) from January 26, 2021, to February 3, 2021. For this cross-sectional study, we estimated a sample size of 2034 participants to assess the prevalence of self-reported ailment (*e.g.* hypertension) at 95% confidence level (1-α) with 2.5% bound on error of estimation assuming a prevalence of self-reported hypertension as 10% in our target population. To account for refusals, and/or partial (incomplete) responses, sample size was inflated to 2500. Furthermore, this sample size was large enough to relate most of the potential exposures (with a prevalence of 0.25 or higher in the general population) and any ailment status with an odds ratio (OR) of 2 or higher using polychotomous logistic regression at a 5% significance level (α) and 85% study power [23].

### Ethics

The research protocol, consent form, and data collection instrument were approved by the Ethics Committee of the Health Sciences Center of Kuwait University (no. 272/ dated 25/1/2021). The online e-questionnaire included a consent form that explained the purpose of the study and clarified that participation is completely voluntary and that they are free to refuse participation without any implication. It also stated that personal information was not required, and data collection was anonymous to ensure the confidentiality of collected information. The consent form was attached with the questionnaire and the participants were requested to sign the consent form before completing the questionnaire. This study was undertaken in accordance with the principles and guidelines of the Declaration of Helsinki for medical research involving human subjects. Furthermore, the methods including design, conduct, data collection, analysis and reporting of the results conformed with the STROBE (Strengthening the Reporting of Observational Studies in Epidemiology) guidelines.

### Data analysis

For all the analyses, SPSS (Statistical Package for Social Sciences) ver. 26 was used. Data collected via Google forms program were converted to a Microsoft Excel file and transferred to an SPSS file for analysis after the exclusion of responses that did not fulfil the inclusion criteria or had inadmissible answers. The descriptive statistics including frequencies (%) of sociodemographics and lifestyle factors were computed. The prevalence (%) of each morbidity in the study sample was computed. The Pearson chi-squared test was used to examine the statistical association between a polychotomous morbidity variable (none, one morbidity or multimorbidity *i.e.,* ≥ 2 morbidities) and sociodemographics and lifestyle factors. Univariable polychotomous logistic regression analysis was conducted to quantify the magnitude of the association between polychotomous morbidity variable and sociodemographic and lifestyle factors that showed statistical significance on the chi-squared analysis. The variables showing statistical significance (*p* ≤ 0.150) on chi-squared and univariable polychotomous logistic regression analyses were considered for inclusion in multivariable polychotomous logistic regression model [24, 25]. A backward stepwise procedure was used to arrive at the final multivariable polychotomous logistic regression model. The likelihood ratio test was used to evaluate the competing models. The variables which showed statistical significance (*p* < 0.05) and a biologically plausible relationship with the outcome variable were retained in the final model. The parameters’ estimates of the final model were used to compute adjusted odds ratios (aOR) and their corresponding 95% confidence intervals (CI) and used for interpretation of the model.

## Results

### Sample characteristics and prevalence of morbidity

Using social media platforms, we distributed 4500 copies of the e-questionnaire to potential participants. A total of 4248 adults (response rate: 94.4%) responded to the e-questionnaire administered through social media platforms. Six hundred and seventy-three respondents were excluded for being non-residents of Kuwait, having invalid answers, or being under 21 years of age. Therefore, data for an effective sample of 3572 (84.1%) respondents were analyzed. The respondents were mainly Kuwaiti (89%) and female (78.3%). The majority were in the range of 21–40 years (66%) or 41 years old or older (34%). Among the respondents, university graduates (77.1%), married (55.2%), and with a total family income of more than 2000 Kuwaiti Dinars (KD) per month (45.5%) were predominant (Table 1).

**Table 1:** Sociodemographic characteristics among adults enrolled in a cross-sectional study of the prevalence of and factors associated with multimorbidity in Kuwait, February 2021 (*N* = 3572)

Characteristic	n	%
Age (completed years)		
21–30	1454	40.7
31–40	902	25.3
41–50	730	20.4
51–60	379	10.6
> 60	107	3.0
Gender		
Male	775	21.7
Female	2797	78.3
Nationality		
Kuwaiti	3180	89.0
Non-Kuwaiti Arab	285	8.0
Non-Kuwaiti, non-Arab	107	3.0
Educational level		
Less than high school	75	2.0
High school	299	8.4
Collage/ University	2754	77.1
Post graduate study	444	12.4
Family income (Kuwaiti Dinars/ month)		
< 500	127	3.6
500–999	430	12.0
1000–1499	707	19.8
1500–2000	683	19.1
> 2000	1625	45.5
Marital status		
Single	1328	37.2
Married	1970	55.2
Divorced	230	6.4
Widowed	44	1.2

**Fig. 1:**
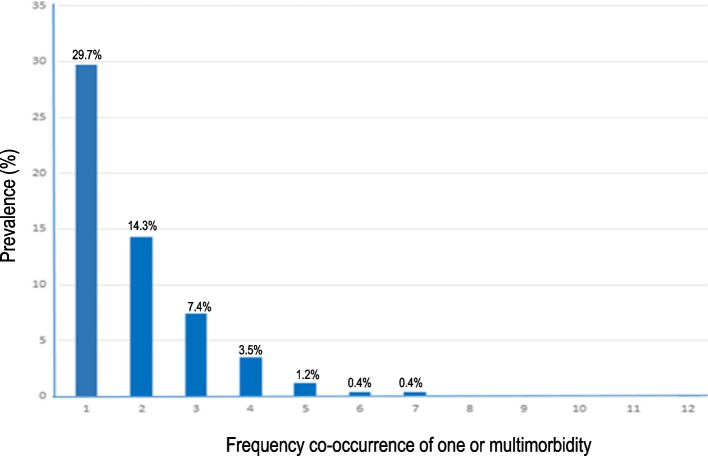
Prevalence (%) of one or more multimorbidity (≥ 2) among adults enrolled in a cross-sectional study, Kuwait, February 2021 (*N* = 3572)

In this study, the highest prevalence of asthma (17.9%) was recorded, followed by osteoarthritis (16.2%), thyroid problems (15.9%), hypertension (15.0%), depression (13.0%), diabetes mellitus (11.4%), osteoporosis (6.3%), cardiovascular disease (5.5%), rheumatism (4.2%), cancer (2.0%), stroke (0.7%), and chronic obstructive pulmonary disease (0.5%). The proportion of adults with no morbidity was 42.7% (Table 2). The overall prevalence of one morbidity and multimorbidity (≥ 2 morbid conditions) was 29.7% and 27.4% respectively. Figure 1 shows the frequencies of the participants with one, two, three, four, or more morbidities.

**Table 2:** Prevalence of each morbidity among adults enrolled in a cross-sectional study, Kuwait, February 2021 (*N* = 3572)

Morbidity (yes vs. no)	n	%
None	1527	42.7
Hypertension	536	15.0
Diabetes mellitus	406	11.4
Cardiovascular disease	197	5.5
Chronic obstructive pulmonary disease (COPD)	19	0.5
Asthma	637	17.9
Osteoarthritis	577	16.2
Rheumatism	150	4.2
Osteoporosis	224	6.3
Cancer	72	2.0
Stroke	24	0.7
Thyroid problems	567	15.9
Depression	464	13.0

**Fig. 2:**
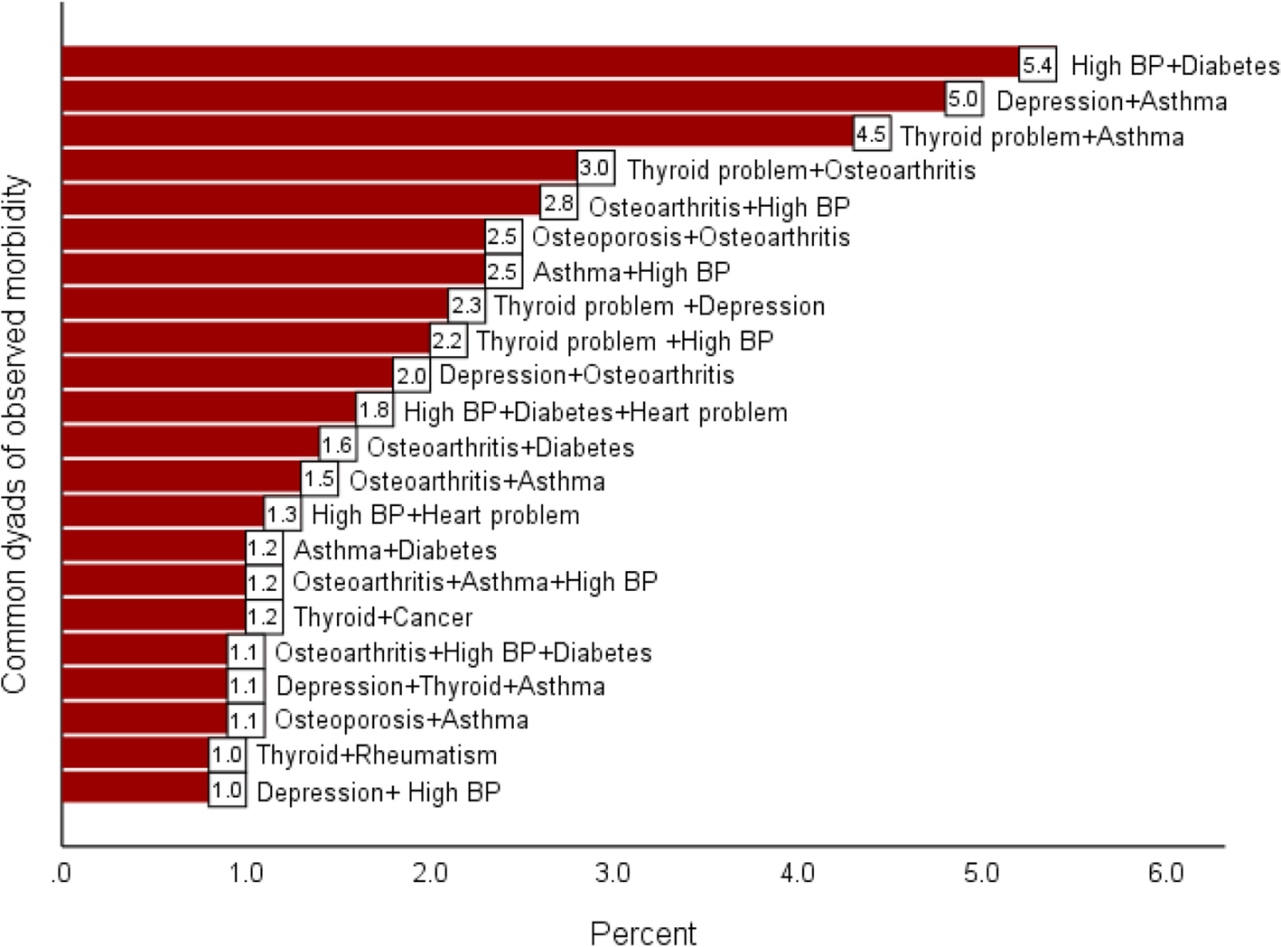
Frequency (%) of common dyads of observed morbidity among adults enrolled in a cross-sectional study, Kuwait, February 2021 (*N* = 3572)

A total of 238 dyads of the assessed conditions were identified, however, the most frequent 18 dyads with a minimum frequency of 1%, are presented in Fig. 2. From the assessed combinations, hypertension combined with diabetes had the highest prevalence (5.4%) followed by depression and asthma (5.0%), thyroid problems and asthma (4.5%). The remaining dyads had a prevalence of 3.0% or less (Fig. 2).

### Univariable associations of sociodemographic and lifestyle factors with morbidity

Chi-squared analysis of morbidity as a polychotomous outcome variable with respect to sociodemographics revealed statistically significant relationship with age (*p* < 0.001), sex (*p* = 0.011), nationality (*p* < 0.001), educational level (*p* < 0.001), combined family income (*p* = 0.010) and marital status (*p* < 0.001). Lifestyle factors statistically significantly related to polychotomous morbidity included smoking status (*p* = 0.007), exposure to secondhand smoke (*p* = 0.048), and physical activity (*p* = 0.001). Whereas alcohol consumption (*p* = 0.057) and sedentary behaviour (*p* = 0.064) had marginally significant (*p* ≥ 0.05 ≤ 0.1) association with the polychotomous outcome variable (the online Supplement Table 1). The distributions of remaining lifestyle and dietary factors are shown in the online Supplement Table 1.

The variables which were significantly related to polychotomous morbidity variable on chi-squared analysis were subjected to univariable polychotomous logistic regression analysis to quantify the magnitude of their association with this polychotomous outcome variable. On univariable polychotomous logistic regression analyses, the demographic variables significantly (*p* < 0.05) or marginally significantly (*p* ≥ 0.05 ≤ 0.1) related to morbidity were age, sex, nationality, education level, combined family income, marital status, smoking status, age at initiation of smoking, exposure to secondhand tobacco smoke, physical activity, and sedentary behaviour (the online Supplement Table 2). The dietary factors including intake of fresh fruit and vegetables (times/ day), frequency of fast-food intake (times per day), fish/seafood intake (times/ week) had a significant but implausible direction of the association with morbidity as a polychotomous outcome, therefore were dropped from further analysis.

### Multivariable polychotomous logistic regression model

A final multivariable polychotomous logistic regression model showed that the demographic factors significantly (*p* < 0.001) associated with one morbid or multimorbid conditions were age and sex (Table 3). As expected, compared with the participants in the age range of 21-30 years, the participants were significantly more likely to have reported one morbidity or multimorbidity if they were in age range of 31-40 years (one morbidity aOR = 1.3, 95%CI: 1.04-1.5; multimorbidity aOR = 2.6, 95% CI: 2.1-3.3), 41-50 years (one morbidity aOR = 2.2, 95%CI: 1.7-2.7; multimorbidity aOR = 6.6, 95%CI: 5.2-8.5), 51-60 years (one morbidity aOR = 3.9, 95%CI: 2.7-5.5; multimorbidity aOR = 25.2, 95%CI: 17.7-35.9) or older than 60 years (one morbidity aOR = 3.6, 95% CI: 1.7-7.7; multimorbidity aOR = 45.5, 95% CI: 23.1-89.5). Compared with the males, the female participants were significantly more likely to have one morbidity (aOR = 1.2, 95% CI: 1.01–1.6) or multimorbidity (aOR = 2.4, 95% CI: 1.9–3.2). Moreover, compared with non-Kuwaiti non-Arabs, there were significantly (*p* < 0.001) increased odds of having multimorbidity among Kuwaiti (aOR = 19.3, 95% CI: 5.8–64.0) and non-Kuwaiti Arab adults (aOR = 14.5, 95% CI: 4.2–50.1). However, on one morbid condition, these three nativity groups did not differ significantly (Table 3).

**Table 3:** Multivariable polychotomous logistic regression analysis of factors associated with physician diagnosed morbidity among adults in Kuwait, February 2021 (*N* = 3572)

**Characteristic**	One morbidity		Multimorbidity		*p*-value
	aOR^a^	95% CI	aOR	95% CI	
**Age (completed years**)					**< 0.001**
21–30	ref	-	ref	-	
31–40	1.3	1.04–1.5	2.6	2.1–3.3	
41–50	2.2	1.7–2.7	6.6	5.2–8.5	
51–60	3.9	2.7–5.5	25.2	17.7–35.9	
> 60	3.6	1.7–7.7	45.5	23.1–89.5	
**Sex**					**< 0.001**
Male	ref	-	ref	-	
Female	1.2	1.01–1.6	2.4	1.9–3.2	
**Nativity**					**< 0.001**
Non-Kuwaiti, non-Arab	ref	-	ref	-	
Kuwaiti	1.1	0.7–1.7	19.3	5.8–64.0	
Non-Kuwaiti Arab	1.1	0.7–1.8	14.5	4.2–50.1	
**Sedentary behavior (hours/day)**					**< 0.001**
1	ref	-	ref	-	
> 1–2	1.4	1.02–2.0	1.1	0.7–1.6	
3–4	1.3	1.0–1.9	1.1	0.8–1.6	
5–6	1.0	0.7–1.5	1.2	0.9–1.8	
> 6	1.4	1.0–2.0	1.6	1.1–2.4	
**Smoking status**					**< 0.001**
Non-Smoker	ref	-	ref	-	
Smoker	0.9	0.6–1.1	1.9	1.4–2.6	
**Ever alcohol drinking**					**0.014**
No	ref	-	ref	-	
Yes	1.6	1.1–2.2	1.6	1.1–2.3	

^a^Adjusted odds ratio

*CI* Confidence interval

Regarding lifestyle factors, adults spending more than six hours in a sedentary routine have had 60% more odds to report multimorbid conditions (aOR = 1.6, 95% CI: 1.1–2.4; *p* < 0.001) compared with those who reportedly have had spent one hour in a similar manner. Likewise, the odds of having multimorbidity among current smokers were 1.9 times the odds of having multimorbidity among non-smokers (aOR = 1.9, 95% CI: 1.4–2.6, *p* < 0.001). Furthermore, alcohol drinkers were significantly (*p* = 0.014) more likely to report one morbidity (aOR = 1.6, 95% CI: 1.1–2.2) or multimorbidity (aOR = 1.6, 95% CI: 1.1–2.3) compared with non-drinkers of alcohol (Table 3).

## Discussion

This cross-sectional study assessed the prevalence of morbidity (one morbidity and multimorbidity) among the adult (21 years old or older) population of Kuwait and examined the association of sociodemographics and lifestyle factors with this polychotomous outcome variable. In this study, the overall prevalence of multimorbidity was 27.4% in the study participants. This estimate is higher compared to the prevalence of multimorbidity reported from the UK (19.6%) [26], and China (11.1%) [10], evaluating 36 and 40 conditions, respectively. However, the assessed prevalence was comparable with that reported from Scotland (23.2%) [9], Brazil (23.6%) [27], Serbia (26.9%) [28], and Cyprus (28.6%) [29]. Similarly, a study in Lebanon among Palestinian refugees reported a 24% prevalence of multimorbidity [30]. However, compared with the prevalence of multimorbidity reported in the present study, some other countries reported higher prevalences, including Australia (32.6%) [31], Indonesia (35.7%) [32], and Canada (59%) [33].

The multivariable polychotomous logistic regression model indicated that age, sex, nativity, sedentary behavior, smoking, and alcohol drinking were significantly and independently associated with multimorbidity. This study showed that with increasing age, the odds of multimorbidity had significant and increasing trends in all age groups compared with the younger age group (21–30 years). Similar age-related trends in one morbidity and multimorbidity had been reported in several studies from various countries across the globe including Cyprus [29], Serbia [28], Brazil [27], and Palestinian refugees [30].

With regards to sex, the odds of one morbidity do not seem to differ significantly between females and males. However, compared with males, the females were 2.4 times more likely to report multimorbidity. This finding was consistent with the results of a study conducted in the Golestan province of Iran, wherein the females compared with males had more than twice the odds of multimorbidity [34, 35]. Similarly, a study in Southern China showed that the odds of multimorbidity in women were 1.7 times the odds of multimorbidity in men [10]. A similar magnitude of association between sex and morbidity (one or two or more) was reported from various other countries, including Scotland [9], Brazil [27], and Indonesia [32]. However, a study in Quebec, Canada reported that sex was not significantly associated with multimorbidity [33]. Interestingly, a cohort study from New Zealand showed a non-significant association between sex and morbidity among subjects aged 50 to 75 years [36]. These sex-related variations in morbidity and multimorbidity might have been reflecting dietary habits and other unmeasured lifestyle patterns.

In this study, compared with the non-Kuwaiti non-Arabs, the Kuwaitis and non-Kuwaiti Arabs were more likely to report multimorbidity. Non-Kuwaiti non-Arabs more often return to their home countries as they get older. Furthermore, Kuwaiti law requires non-Kuwaitis aged 70 years or above to return to their countries; therefore, possibly over-representation of Kuwaitis and non-Kuwaiti Arabs in the sample might have led to the observed association. However, the adjusted association between nativity and one-morbidity was statistically non-significant.

This study showed that spending more than six hours in a sedentary manner resulted in a 60% increase in the odds of reporting multimorbidity in adults, compared with those spending only an hour in a similar manner. This was evident in a study among adults (> 50 years old) in low-and middle-income countries, where the prevalence of sedentary behavior was 7.1% in people with no chronic conditions vs. 24.1% in those with more than four chronic conditions [37, 38]. Furthermore, a study from the USA reported an 11% increase in the odds of reporting multimorbidity with every 60-min increase in sedentary behavior [39]. From another perspective, an Irish study has also established a positive association between the individual chronic diseases count and the daily average number of minutes in sedentary behavior [40].

In the present study, the odds of one-morbidity or odds of multimorbidity in either case were 1.6 times higher in those who reportedly were regular alcohol drinkers at any point in time compared with those who never drank alcohol. Likewise, the current smokers had 1.9 times higher odds of reporting multimorbidity than those of the non-smokers. However, for one morbidity, smokers and non-smokers did not differ significantly. These results are congruent with the findings of a study from Southern China, which reported significant and independent associations between multimorbidity and alcohol drinking (odds ratio 3.5) and smoking (odds ratio 3.1) [10]. Additionally, An Iranian study also reported a significant association between alcohol drinking and multimorbidity from Iran [35], and Botswana (odds ratio 4.8) [41]. Another longitudinal Finnish study has demonstrated a significant positive association between smoking and multimorbidity in both men (hazard ratio 2.7) and women (hazard ratio 2.5) [42]. As opposed to our results, a study in New Zealand showed that regular alcohol consumption was protective against developing multimorbidity (odds ratio 0.8) and a statistically non-significant association between smoking and multimorbidity [36]. Nevertheless, overall, the results of this study and anecdotal evidence flag high risk of multimorbidity associated with smoking and alcohol drinking which entails the public health intervention.

The current study showed that the most prevalent combinations of morbidities were first hypertension and diabetes, then depression and asthma, followed by thyroid problems and asthma. These findings cannot be accurately compared with those reported by other studies since generated clusters of morbidities differ; however, the broader categories of the assessed clusters were comparable. As the results of this study showed that cardiometabolic conditions were the most prevalent combinations which were also demonstrated by many other studies as well [27, 28, 31, 42, 43]. Additionally, psychotic, respiratory, and endocrine conditions were found to be highly prevalent, which is in agreement the published literature [37, 38]. The combination of hypertension and diabetes was prevalent in 5.4% of the study participants in this study. This combination is expected since diabetes contributes to the development of hypertension by increasing vascular stiffness and therefore raising the systolic blood pressure. Moreover, hyperglycemia-induced increase of filtered glucose increases sodium retention, leading to volume expansion and a rise in blood pressure [44].

This study reported asthma and depression as the second most prevalent combination. This finding draws special attention since asthma is mainly a disease of the young individuals and it is already established to be a major problem in Kuwait [45, 46]. This association with depression is alarming and requires a closer evaluation of asthmatic patients and their quality of life.

This research showed thyroid problems to be the third most prevalent morbidity (15.9%) and presents in 4 of the 10 most common reported dyads perhaps owing to the fact that more than half of the respondents were aged less than 40 years and 78.3% were females. The reason for such a high burden of thyroid problems in Kuwait is an important topic for further research.

Multimorbid patients are more prone to experience fragmentation of care and medical error due to the shifted focus from the patient as a whole to the individual diseases they exhibit, and this is a major challenge for healthcare systems worldwide [9]. The rapid increase in life expectancy will only increment the demand on healthcare systems to provide for a larger number of patients with multimorbidity; therefore, it would be beneficial if a nation-wide public health exploration is implemented to document the incidence, prevalence, and specific risk factors associated with the condition at hand. Public health professionals could also organize campaigns targeted at increasing public awareness regarding physical inactivity and tobacco smoking and their indefinite association with most morbidities. However, more research is needed to examine the temporal link between multimorbidity and different risk factors.

### Study strengths and limitations

The key strength of this research is that it is one of the pioneering studies on the prevalence of multimorbidity among the adult population of Kuwait, providing a basis for future research. The findings of this study showed an evident pattern in the co-occurrence of chronic non-communicable diseases and their associated factors in the country.

Some limitations of this study need to be taken into consideration; *first*, this being a cross-sectional study, the temporal link of the studied factors with multimorbidity could not be established; therefore, the observed associations may not truly be causal in nature. *Second,* our sample of participants could not be considered representative of the adult population of Kuwait because most participants were Kuwaiti and young adults; therefore, the generalizability of results beyond the study sample needs to be appraised carefully. *Third,* inadvertently we missed out obesity from inclusion in the questionnaire, since in the context of Kuwait, it is an important health abnormality. It would have been interesting to see obesity in conjunction with other morbid conditions. Future studies may consider it for inclusion in the study instrument. *Fourth,* we did not ascertain the severity of the reported morbidities. *Fifth,* the comparison of multimorbidity prevalence across different regions must be interpreted with caution due to the heterogeneity of the study designs in terms of population, methods, and measured diseases. *Sixth*, for the purpose of quality assurance, we could not include inbuilt validity checks for the collected data, therefore future studies may contemplate this aspect at the planning stage. *Finally*, there is no standard methodology of measuring multimorbidity leading to heterogeneity in literature, making it difficult to compare studies efficiently.

## Conclusions

This study showed a high prevalence of one-morbidity, multimorbidity, and specific combinations of various morbidities. We identified critical demographics and lifestyle factors associated with one-morbidity or multimorbidity, including age, sex, nativity, sedentary behavior, smoking, and alcohol drinking. Based on the identified lifestyle factors, public health authorities may consider instituting educational interventions to minimize the prevalence of these modifiable risk factors. If implemented, future studies may evaluate the impact of such interventions.

### Supplementary Information

**Supplementary Material 1.**

**Supplementary Material 2.**

## Data Availability

On a reasonable request the data used in the study can be made available by the corresponding author.
