# Supplementary material for: Prevalence of and factors associated with multimorbidity among adults in Kuwait
Source: BMC Public Health. 2024 Mar 12;24:768. doi: 10.1186/s12889-024-18298-z (PMC10936001; doi:10.1186/s12889-024-18298-z)
Supplement: Supplementary file 1 — Supplementary Material 1. [file 12889_2024_18298_MOESM1_ESM.docx]

**Supplementary Table 1.** Chi-squared analysis of the relationship between sociodemographic, lifestyle,

dietary factors, and morbidity among adults in Kuwait, February 2021 (N = 3572)

| Variable | Category total | No morbidity  n (%) | One morbidity  n (%) | Multimorbidity^[[1]](#footnote-1)^  n (%) | *p*-value* |
| --- | --- | --- | --- | --- | --- |
| ***Sociodemographic factors*** | | | | | |
| Age (completed years) |  |  |  |  | < 0.001 |
| 21-30 | 1454 | 833 (57.3) | 431 (29.6) | 190 (13.1) |  |
| 31-40 | 902 | 411 (45.6) | 269 (29.8) | 222 (24.6) |  |
| 41-50 | 730 | 215 (29.5) | 236 (32.3) | 279 (38.2) |  |
| 51-60 | 379 | 57 (15.0) | 106 (28.0) | 216 (57.0) |  |
| > 60 | 107 | 11 (10.3) | 20 (18.7) | 76 (71.0) |  |
| Gender |  |  |  |  | 0.011 |
| Male | 775 | 357 (46.1) | 237 (30.6) | 181 (23.4) |  |
| Female | 2797 | 1170 (41.8) | 825 (29.5) | 802 (28.7) |  |
| Nationality |  |  |  |  | < 0.001 |
| Kuwaiti | 3180 | 1335 (42.0) | 926 (29.1) | 919 (28.9) |  |
| Non-Kuwaiti Arab | 285 | 136 (47.7) | 88 (30.9) | 61 (21.4) |  |
| Non-Kuwaiti non-Arab | 107 | 56 (52.3) | 48 (44.9) | 3 (2.8) |  |
| Educational level |  |  |  |  | < 0.001 |
| Less than high school | 75 | 28 (37.3) | 20 (26.7) | 27 (36.0) |  |
| High school | 299 | 104 (34.8) | 80 (26.8) | 115 (38.5) |  |
| College/ university,  postgraduate | 3198 | 1395 (43.6) | 962 (30.1) | 841 (26.3) |  |

| Combined family income (KD/ months) |  |  |  |  | 0.010 |
| --- | --- | --- | --- | --- | --- |
| < 500 | 127 | 65 (51.2) | 38 (29.9) | 24 (18.9) |  |
| 500-999 | 430 | 177 (41.2) | 118 (27.4) | 135 (31.4) |  |
| 1000-1499 | 707 | 326 (46.1) | 215 (30.4) | 166 (23.5) |  |
| 1500-2000 | 683 | 270 (39.5) | 201 (29.4) | 212 (31.0) |  |
| > 2000 | 1625 | 689 (42.4) | 490 (30.2) | 446 (27.4) |  |
| Marital status |  |  |  |  | < 0.001 |
| Single | 1328 | 690 (52.0) | 404 (30.4) | 234 (17.6) |  |
| Married | 1970 | 757 (38.4) | 585 (29.7) | 628 (31.9) |  |
| Divorced | 230 | 74 (32.2) | 68 (29.6) | 88 (38.3) |  |
| Widowed | 44 | 6 (13.6) | 5 (11.4) | 33 (75.0) |  |

| ***Lifestyle factors*** | | | | | |
| --- | --- | --- | --- | --- | --- |
| Smoking status |  |  |  |  | 0.007 |
| Smoker | 467 | 196 (42.0) | 117 (25.1) | 154 (33.0) |  |
| Nonsmoker | 3105 | 1331 (42.9) | 945 (30.4) | 829 (26.7) |  |
| Smoking intensity^[[2]](#footnote-2)^ |  |  |  |  | 0.247 |
| Non-smokers | 3008 | 1277 (42.5) | 913 (30.4) | 818 (27.2) |  |
| Light smoker | 174 | 73 (42.0) | 45 (25.9) | 56 (32.2) |  |
| Moderate smoker | 161 | 80 (49.7) | 40 (24.8) | 41 (25.5) |  |
| Heavy smoker | 130 | 54 (41.5) | 34 (26.2) | 42 (32.3) |  |
| Smoking onset |  |  |  |  | 0.010 |
| Do not smoke | 3008 | 1277 (42.5) | 913 (30.4) | 818 (27.2) |  |
| Before age of 10 | 63 | 38 (60.3) | 13 (20.6) | 12 (19.0) |  |
| During age of 10-14 | 87 | 42 (48.3) | 18 (20.7) | 27 (31.0) |  |
| At the age of 15 | 361 | 142 (39.3) | 101 (28.0) | 118 (32.7) |  |
| Secondhand smoke exposure |  |  |  |  | 0.048 |
| Exposed | 2432 | 1029 (42.3) | 704 (28.9) | 699 (28.7) |  |
| Not exposed | 1140 | 498 (43.7) | 358 (31.4) | 284 (24.9) |  |
| Smoking method^[[3]](#footnote-3)^ |  |  |  |  | 0.596** |
| Cigarettes only | 160 | 76 (47.5) | 37 (23.1) | 47 (29.4) |  |
| Cigarettes and hookah | 134 | 61 (45.5) | 34 (25.4) | 39 (29.1) |  |
| Cigarettes and Electronic-Cigarettes | 212 | 83 (39.2) | 58 (27.4) | 71 (33.5) |  |
| Cigarettes and pipe smoking | 8 | 4 (50.0) | 3 (37.5) | 1 (12.5) |  |
| Physical activity^[[4]](#footnote-4)^ |  |  |  |  | 0.001 |
| Insufficient | 2512 | 1065 (42.4) | 713 (28.4) | 734 (29.2) |  |
| Sufficient | 1060 | 462 (43.6) | 349 (32.9) | 249 (23.5) |  |
| Sedentary behavior (hrs/day) |  |  |  |  | 0.064 |
| 1 | 283 | 126 (44.5) | 76 (26.9) | 81 (28.6) |  |
| 1-2 | 627 | 244 (38.9) | 213 (34.0) | 170 (27.1) |  |
| 3-4 | 982 | 417 (42.5) | 307 (31.3) | 258 (26.3) |  |
| 5-6 | 711 | 329 (46.3) | 183 (25.7) | 199 (28.0) |  |
| >6 | 969 | 411 (42.4) | 283 (29.2) | 275 (28.4) |  |
| Fresh vegetables intake (time/day) |  |  |  |  | < 0.001 |
| 0 | 359 | 185 (51.5) | 104 (29.0) | 70 (19.5) |  |
| 1-2 | 1384 | 628 (45.4) | 378 (27.3) | 378 (27.3) |  |
| 3-4 | 922 | 359 (38.9) | 294 (31.9) | 269 (29.2) |  |
| ≥5 | 907 | 355 (39.1) | 286 (31.5) | 266 (29.3) |  |
| Fresh fruit intake (time(s)/day) |  |  |  |  | < 0.001 |
| 0 | 590 | 277 (46.9) | 178 (30.2) | 135 (22.9) |  |
| 1-2 | 1603 | 707 (44.1) | 454 (28.3) | 442 (27.6) |  |
| 3-4 | 795 | 339 (42.6) | 225 (28.3) | 231 (29.1) |  |
| ≥5 | 584 | 204 (34.9) | 205 (35.1) | 175 (30.0) |  |
| Fast food intake (time(s)/day) |  |  |  |  | < 0.001 |
| 0 | 968 | 351 (36.3) | 295 (30.5) | 322 (33.3) |  |
| 1-2 | 1712 | 727 (42.5) | 518 (30.3) | 467 (27.3) |  |
| 3-4 | 626 | 314 (50.2) | 176 (28.1) | 136 (21.7) |  |
| ≥5 | 266 | 135 (50.8) | 73 (27.4) | 58 (21.8) |  |
| Fish /seafood intake (day(s)/week) |  |  |  |  | 0.007 |
| 0 | 1310 | 579 (44.2) | 385 (29.4) | 346 (26.4) |  |
| 1-2 | 1949 | 828 (42.5) | 573 (29.4) | 548 (28.1) |  |
| 3-4 | 274 | 95 (34.7) | 94 (34.3) | 85 (31.0) |  |
| ≥5 | 39 | 25 (64.1) | 10 (25.6) | 4 (10.3) |  |

| Red meat intake (day(s)/week) |  |  |  |  | 0.337 |
| --- | --- | --- | --- | --- | --- |
| 0 | 661 | 283 (42.8) | 191 (28.9) | 187 (28.3) |  |
| 1-2 | 1935 | 810 (41.9) | 569 (29.4) | 556 (28.7) |  |
| 3-4 | 783 | 348 (44.4) | 238 (30.4) | 197 (25.2) |  |
| ≥5 | 193 | 86 (44.6) | 64 (33.2) | 43 (22.3) |  |
| Ever alcohol consumption |  |  |  |  | 0.057 |
| Yes | 253 | 90 (35.6) | 85 (33.6) | 78 (30.8) |  |
| No | 3319 | 1437 (43.3) | 977 (29.4) | 905 (27.3) |  |
| 12-month alcohol abstinence^[[5]](#footnote-5)^ |  |  |  |  | 0.235 |
| Yes | 116 | 44 (37.9) | 42 (36.2) | 30 (25.9) |  |
| No | 134 | 43 (32.1) | 43 (32.1) | 48 (35.8) |  |
| Current alcohol consumption^[[6]](#footnote-6)^ |  |  |  |  | 0.514 |
| Yes | 63 | 24 (38.1) | 24 (83.1) | 15 (23.8) |  |
| No | 145 | 54 (37.2) | 46 (31.7) | 45 (31.0) |  |

1. Definition: ≥ 2 morbidities.

   * *p*-values for Pearson Chi-squared test statistic unless specified otherwise (α = 0.05). [↑](#footnote-ref-1)
2. Definition: light ≤10 cigarettes, Moderate 20-40 cigarettes, heavy ≥40 cigarettes (Kaleta, 2012). [↑](#footnote-ref-2)
3. Frequencies among smokers; values will not add up to the total sample size. [↑](#footnote-ref-3)
4. Defined according to WHO recommendations on physical activity: insufficient < 150 minutes of moderate-intensity activity per week (WHO, 2020)

   ** *p-*value of likelihood ratio [↑](#footnote-ref-4)
5. Values recorded are among subjects ever drank, so will not add up to the total sample size. [↑](#footnote-ref-5)
6. Defined according to WHO as drinking alcohol in the past 30 days (MOH, 2015). Values are among subjects ever drank [↑](#footnote-ref-6)
