# Supplementary material for: Prevalence of and factors associated with multimorbidity among adults in Kuwait
Source: BMC Public Health. 2024 Mar 12;24:768. doi: 10.1186/s12889-024-18298-z (PMC10936001; doi:10.1186/s12889-024-18298-z)
Supplement: Supplementary file 2 — Supplementary Material 2. [file 12889_2024_18298_MOESM2_ESM.docx]

| \| **Supplementary Table 2.** Univariable polychotomous logistic regression analysis of factors associated with physician diagnosed morbidity among adults in Kuwait, February 2021 (N = 3572) \| \| \| \| \| \| \| --- \| --- \| --- \| --- \| --- \| --- \| \| **Characteristic** \| One morbidity \| \| Multimorbidity \| \| *p-*value \| \|  \| OR \| 95% CI \| OR \| 95% CI \|  \| \| Age (completed years) < 0.001 \| \| \| \| \| \| \| 21-30 \| ref \| - \| ref \| - \|  \| \| 31-40 \| 1.3 \| 1.0-1.5 \| 2.4 \| 1.9-3.0 \|  \| \| 41-50 \| 2.1 \| 1.7-2.6 \| 5.7 \| 4.5-7.2 \|  \| \| 51-60 \| 3.6 \| 2.5-5.1 \| 16.6 \| 11.9-23.1 \|  \| \| > 60 \| 3.5 \| 1.7-7.4 \| 30.3 \| 15.8-58.1 \|  \| \| Gender 0.010 \| \| \| \| \| \| \| Male \| ref \| - \| ref \| - \|  \| \| Female \| 1.1 \| 0.9 -1.3 \| 1.4 \| 1.1-1.7 \|  \| \| Nationality < 0.001 \| \| \| \| \| \| \| Non-Kuwaiti, non-Arab \| ref \| - \| ref \| - \|  \| \| Kuwaiti \| 0.8 \| 0.5-1.2 \| 12.9 \| 4.0-41.2 \|  \| \| Non-Kuwaiti Arab \| 0.8 \| 0.5-1.2 \| 8.4 \| 2.5-27.8 \|  \| \| Education level < 0.001 \| \| \| \| \| \| \| Collage/ University/ Postgraduate \| ref \| - \| ref \| - \|  \| \| Uneducated/Primary/Intermediate \| 1.0 \| 0.6-1.8 \| 1.6 \| 0.9-2.7 \|  \| \| High school \| 1.1 \| 0.8-1.5 \| 1.8 \| 1.4-2.4 \|  \| \| Combined Family Income (Kuwaiti Dinars/ month) 0.009 \| \| \| \| \| \| \| < 500 \| ref \| - \| ref \| - \|  \| \| 500-999 \| 1.1 \| 0.7-1.8 \| 2.1 \| 1.2-3.5 \|  \| \| 1000-1499 \| 1.1 \| 0.7-1.7 \| 1.4 \| 0.8-2.3 \|  \| \| 1500-2000 \| 1.3 \| 0.8-2.0 \| 2.1 \| 1.3-3.5 \|  \| \| > 2000 \| 1.2 \| 0.8-1.8 \| 1.8 \| 1.1-2.8 \|  \| \| Marital status < 0.001 \| \| \| \| \| \| \| Single \| ref \| - \| ref \| - \|  \| \| Married \| 1.3 \| 1.1-1.5 \| 2.4 \| 2.0-2.9 \|  \| \| Divorced \| 1.5 \| 1.1-2.2 \| 3.5 \| 2.4-4.9 \|  \| \| Widowed \| 1.4 \| 0.4-4.6 \| 16.2 \| 6.7-39.1 \|  \| \| Smoking 0.008 \| \| \| \| \| \| \| Non-smoker \| ref \| - \| ref \| - \|  \| \| Smoker \| 0.8 \| 0.7-1.1 \| 1.3 \| 1.0-1.6 \|  \| \| Age at smoking initiation 0.011 \| \| \| \| \| \| \| I don’t smoke \| ref \| - \| ref \| - \|  \| \| Before the age of 10 \| 0.5 \| 0.3-0.9 \| 0.5 \| 0.3-.09 \|  \| \| During age of 10-14 \| 0.6 \| 0.3-1.0 \| 1.0 \| 0.6-1.6 \|  \| \| At the age of 15 \| 1.0 \| 0.8-1.3 \| 1.3 \| 1.0-1.7 \|  \| \| **Secondhand smoke exposure** 0.047 \| \| \| \| \| \| \| Not exposed to smoking \| ref \| - \| ref \| - \|  \| \| Exposed to smoking \| 1.0 \| 0.8-1.1 \| 1.2 \| 1.0-1.4 \|  \| \| Physical Activity 0.001 \| \| \| \| \| \| \| Insufficient \| 0.9 \| 0.7-1.0 \| 1.3 \| 1.1-1.5 \|  \| \| Sufficient \| ref \| - \|  \| - \|  \| \| Sedentary Behavior (hours/day) 0.063 \| \| \| \| \| \| \| 1 \| ref \| - \| ref \| - \|  \| \| >1-2 \| 1.4 \| 1.0-2.0 \| 1.1 \| 0.8-1.5 \|  \| \| 3-4 \| 1.2 \| 0.9-1.7 \| 1.0 \| 0.7-1.3 \|  \| \| 5-6 \| 0.9 \| 0.7-1.3 \| 0.9 \| 0.7-1.3 \|  \| \| > 6 \| 1.1 \| 0.8-1.6 \| 1.0 \| 0.8-1.4 \|  \| \| Ever Alcohol Consumption 0.054 \| \| \| \| \| \| \| No \| ref \| - \| ref \| - \|  \| \| Yes \| 1.4 \| 1.0-1.9 \| 1.4 \| 1.0-1.9 \|  \| |
| --- | --- | --- | --- | --- | --- | --- | --- | --- | --- | --- | --- | --- | --- | --- | --- | --- | --- | --- | --- | --- | --- | --- | --- | --- | --- | --- | --- | --- | --- | --- | --- | --- | --- | --- | --- | --- | --- | --- | --- | --- | --- | --- | --- | --- | --- | --- | --- | --- | --- | --- | --- | --- | --- | --- | --- | --- | --- | --- | --- | --- | --- | --- | --- | --- | --- | --- | --- | --- | --- | --- | --- | --- | --- | --- | --- | --- | --- | --- | --- | --- | --- | --- | --- | --- | --- | --- | --- | --- | --- | --- | --- | --- | --- | --- | --- | --- | --- | --- | --- | --- | --- | --- | --- | --- | --- | --- | --- | --- | --- | --- | --- | --- | --- | --- | --- | --- | --- | --- | --- | --- | --- | --- | --- | --- | --- | --- | --- | --- | --- | --- | --- | --- | --- | --- | --- | --- | --- | --- | --- | --- | --- | --- | --- | --- | --- | --- | --- | --- | --- | --- | --- | --- | --- | --- | --- | --- | --- | --- | --- | --- | --- | --- | --- | --- | --- | --- | --- | --- | --- | --- | --- | --- | --- | --- | --- | --- | --- | --- | --- | --- | --- | --- | --- | --- | --- | --- | --- | --- | --- | --- | --- | --- | --- | --- | --- | --- | --- | --- | --- | --- | --- | --- | --- | --- | --- | --- | --- | --- | --- | --- | --- | --- | --- | --- | --- | --- | --- | --- | --- | --- | --- | --- | --- | --- | --- | --- | --- | --- | --- | --- | --- | --- | --- | --- | --- | --- | --- | --- | --- | --- | --- | --- | --- | --- | --- | --- | --- | --- | --- | --- | --- | --- | --- | --- | --- | --- | --- | --- | --- | --- | --- | --- | --- | --- | --- | --- | --- | --- | --- | --- | --- | --- | --- | --- | --- | --- | --- | --- | --- | --- | --- | --- | --- | --- | --- | --- | --- | --- | --- | --- | --- | --- | --- | --- | --- | --- | --- | --- | --- | --- | --- | --- | --- | --- | --- | --- | --- | --- | --- | --- | --- | --- | --- | --- | --- | --- | --- | --- | --- | --- | --- | --- | --- | --- |

Note: Intake of four dietary item including fresh fruit, fresh vegetables, fast food, and fish/ seafood

consumption had significant but implausible direction of association with polychotomous morbidity.

outcome variable. Therefore, these four variable data are not shown in T able 3. Also, none of these

variables were significant in multivariable analysis.
